# A model for supporting biomedical and public health researcher use of publicly available *All of Us* data at Historically Black Colleges and Universities

**DOI:** 10.1093/jamia/ocae099

**Published:** 2024-05-10

**Authors:** Brian Southwell, Sula Hood, Javan Carter, Courtney Richardson, Sheri Cates, Hadyatoullaye Sow, MaryBeth Branigan, Trey-Rashad Hawkins, Katie Atkinson, Jennifer Uhrig, Megan Lewis

**Affiliations:** Social, Statistical, and Environmental Sciences, RTI International, Research Triangle Park, NC 27709, United States; Social, Statistical, and Environmental Sciences, RTI International, Research Triangle Park, NC 27709, United States; Social, Statistical, and Environmental Sciences, RTI International, Research Triangle Park, NC 27709, United States; Social, Statistical, and Environmental Sciences, RTI International, Research Triangle Park, NC 27709, United States; Social, Statistical, and Environmental Sciences, RTI International, Research Triangle Park, NC 27709, United States; Social, Statistical, and Environmental Sciences, RTI International, Research Triangle Park, NC 27709, United States; Social, Statistical, and Environmental Sciences, RTI International, Research Triangle Park, NC 27709, United States; Social, Statistical, and Environmental Sciences, RTI International, Research Triangle Park, NC 27709, United States; Social, Statistical, and Environmental Sciences, RTI International, Research Triangle Park, NC 27709, United States; Social, Statistical, and Environmental Sciences, RTI International, Research Triangle Park, NC 27709, United States; Social, Statistical, and Environmental Sciences, RTI International, Research Triangle Park, NC 27709, United States

**Keywords:** health inequity, biomedical, precision medicine, teaching, communication program

## Abstract

**Purpose:**

The aim of this study was to describe opportunities and challenges associated with the development and implementation of a program for supporting researchers underrepresented in biomedical research.

**Approach:**

We describe a case study of the *All of Us* Researcher Academy supported by the National Institutes of Health (NIH), including feedback from participants, instructors, and coaches.

**Findings:**

Lessons include the importance of inviting role models into learning networks, establishing and maintaining trusted relationships, and making coaches available for technical questions from researcher participants.

**Originality:**

Although research has focused on learning outcomes in science, technology, engineering, and mathematics at Minority Serving Institutions in the United States, literature tends to lack models for initiatives to improve everyday research experiences of faculty and researchers at such institutions or to encourage researcher use of public-use data such as that available through NIH’s *All of Us* Research Program. The *All of Us* Researcher Academy offers a model that addresses these needs.

Inequity between demographic groups in learning and professional training related to science, technology, engineering, and mathematics (STEM) has challenged the advancement of scientific knowledge in the United States for decades.^1^ Black and Hispanic workers in the United States are underrepresented in the STEM workforce relative to population distribution, for example, and Black and Hispanic adults also constitute a lower percentage of STEM graduates from higher education institutions than population distribution would predict.^2^^,^^3^ Such trends suggest that we will continue to see inequities in contributions to STEM without substantial intervention.

Considerable research has assessed student learning outcomes in STEM at Minority Serving Institutions (MSIs) such as Historically Black Colleges and Universities (HBCUs).^4^ We have evidence of population-level demographic differences in perceptions about topics such as precision medicine.^5^ The available literature, however, is more limited regarding the everyday research experiences of faculty and researchers at institutions such as HBCUs. Research to understand HBCU-affiliated researchers’ experiences nonetheless has highlighted the importance of having access to supportive professional networks,^6^ opportunities to participate in grant-review processes,^7^ and mentoring for research publication development and professional resilience.^8^ Promoting STEM research by researchers underrepresented in biomedical research requires more than the availability of grant money.

Developing programs to support researchers affiliated with institutions such as HBCUs could help to translate available grant resources into innovative biomedical research approaches and health science advancements. HBCUs have played an important role in the history of the United States, having been open to people of all backgrounds even when other institutions had more restrictive admissions policies centered on the exclusion of Black students.^9^ The Higher Education Act of 1965 explicitly defined HBCUs as institutions established prior to 1964 that had a principal mission to serve Black Americans, citizens who were systematically denied admission elsewhere. HBCUs today are a vibrant part of the U.S. higher education landscape.

## The *All of Us* Researcher Academy approach

To redress insufficient diversity among research participants in biomedical research in the United States, the National Institutes of Health (NIH) has developed a large-scale data collection effort branded as the *All of Us* Research Program. *All of Us* is designed to build one of the largest, most diverse health databases of its kind, engaging at least 1 million participants who reflect the diversity of the United States. Data contributed by participants could help drive innovation in health care and health research.

Although the *All of Us* dataset offers publicly available data, whether researchers of various backgrounds would publish research using *All of Us* data without additional resources has not been clear. In spring 2022, RTI International worked with NIH to develop the *All of Us* Researcher Academy. The goal of the *All of Us* Researcher Academy has been to increase diversity in the array of researchers actively using *All of Us* data by encouraging and supporting students, postdoctoral researchers, and faculty at institutions with a track record of supporting people underrepresented in biomedical research.

**Figure 1.**
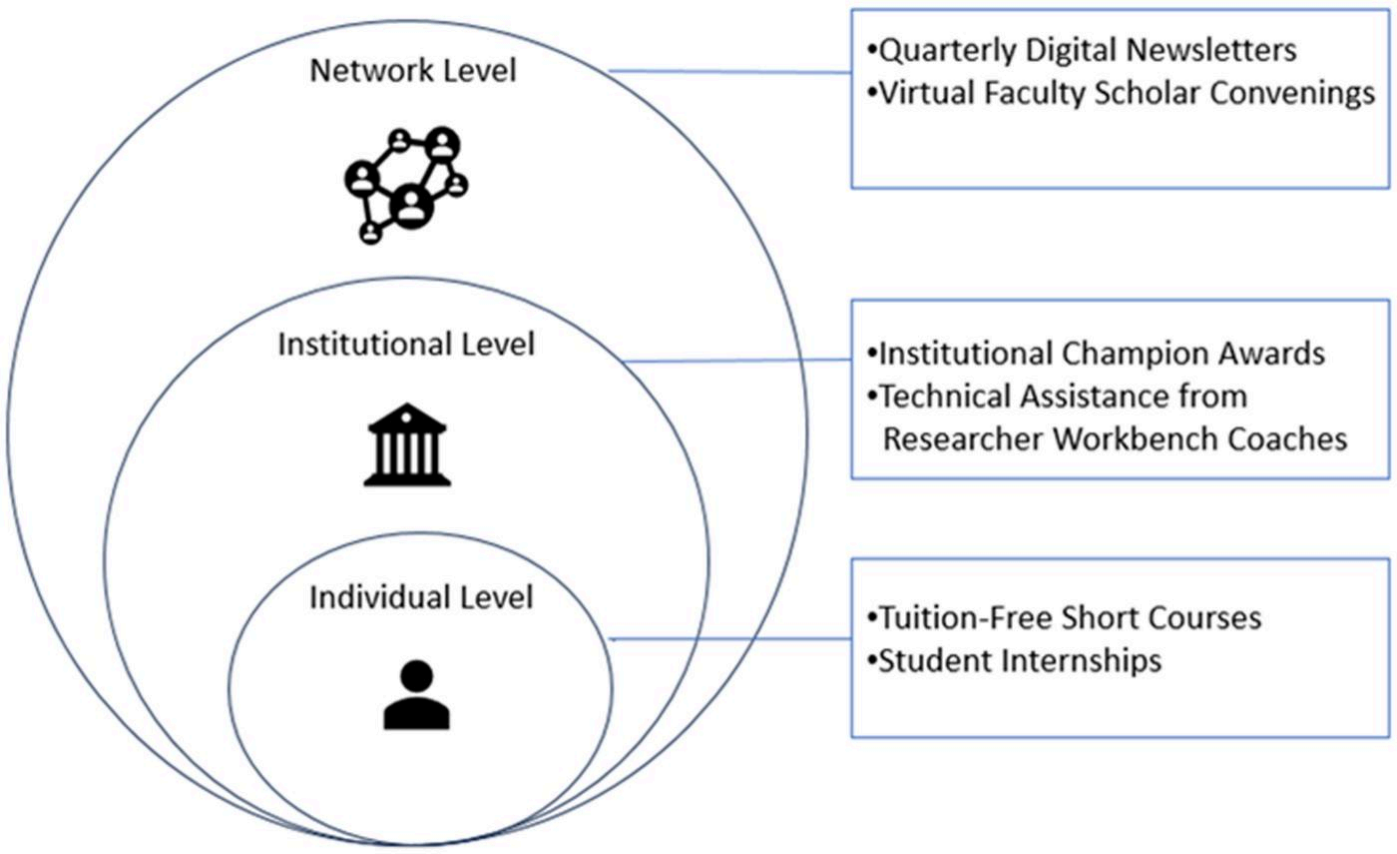
The multi-level structure of the *All of Us* Researcher Academy.

We designed the *All of Us* Researcher Academy to be more than just a clearinghouse of training materials or a grant program providing stipends to researchers. Instead, we developed a multi-level programmatic approach including 6 types of resources for participants. Figure 1 presents a conceptual framework for the *All of Us* Researcher Academy. We designed the Academy to build connections between researchers by establishing a learning network comprising faculty, postdoctoral researchers, and students. The *All of Us* Academy provides capacity building, training, technical assistance, and peer-to-peer learning for health researchers using the *All of Us* Researcher Workbench. Year one of the *All of Us* Researcher Academy focused on HBCU-affiliated researchers.

### Individual support: tuition-free courses and workshops

To provide technical skills and conceptual guidance to encourage biomedical research by researchers affiliated with MSIs, we developed a series of synchronous and asynchronous online courses. Most Academy courses have focused on skills necessary to use the *All of Us* data set for publishable peer-reviewed research, including the use of R for data analysis, data visualization, and coding with Python for data organization. Some courses also have explored considerations for career advancement, including grant-writing advice, publication preparation, and discussion of the peer review process with *Nature* journal staff. Table 1 describes courses offered during year 1 of the *All of Us* Researcher Academy.

**Table 1.** Course implementation and outcomes in year 1.

Course name	Number registered	Average score on perceived learning in course (on 5-point scale, with 5 being the highest response)
Introduction to R and Data Visualization	18	4.35 (*n* = 17)
Introduction to Publishing Health Data in Academic Journals	23	4.36 (*n* = 11)
Writing Your First NIH Grant	10	4.71 (*n* = 7)
Mindful Approaches for Stress Management for Health Researchers	7	4.75 (*n* = 4)
Mindfulness and Coping with Microaggressions for Health Researchers	6	4.25 (*n* = 4)
Advanced Grant Writing for NIH Applications	12	4.6 (*n* = 5)
Introduction to Data Wrangling and Analysis Using R	33	4.6 (*n* = 10)
Introduction to Quantitative Intersectionality Research	19	4.38 (*n* = 8)
Introduction to Python and Pandas for Analysis of *All of Us* Data	20	3.5 (*n* = 2)
Being an Effective Peer Reviewer (*Nature*)	22	5.0 (*n* = 1)
Advanced Topics in Publishing Health Data: Promoting Your Publications	27	5.0 (*n* = 6)

### Individual support: internship program

Academy staff established an internship program for HBCU students in 2023, offering a mentored experiential opportunity for undergraduate and graduate students. Interns work with researchers who use the *All of Us* Researcher Workbench to answer questions about human health and wellbeing with *All of Us* data.

### Institutional support: Institutional Champion awards

Central to the Academy have been teams who responded to an invitation to apply for an Institutional Champion (IC) award (issued publicly as a Request for Applications). The awards included financial support for institutional teams, members of which committed to enrolling in free courses offered by the Academy or mentoring students on projects involving the *All of Us* dataset. In 2022, we announced a first group of Institutional Champions: Howard University, Morgan State University, North Carolina A&T State University, North Carolina Central University, Southern University at New Orleans, and Tuskegee University. Researchers on IC teams investigated topics such as social determinants of health and cardiometabolic risk, breast cancer disparities, mental health equity, and genetic risk in individuals with chronic kidney disease. In 2023, we announced a second group of ICs, including Fayetteville State University, Fisk University, Jackson State University (including researchers from Coahoma Community College), Meharry Medical College, Morehouse School of Medicine, and Shaw University.

IC participants enrolled in tuition-free, online courses and connected with participants at other institutions through those courses, which were free to all researchers in the United States. In addition, Academy staff organized in-person events and webinars highlighting research opportunities using *All of Us* data, including events at Howard University and Tuskegee University organized by the nonprofit organization Community-Campus Partnerships for Health. Researchers at 40 different HBCUs participated in *All of Us* Researcher Academy events in the first year of the Academy.

### Institutional support: researcher workbench coaches

Course instructors played a key role in offering course content, but we also developed Academy coaching roles to support IC teams during and after the courses. Coaches interacted with IC participants outside of courses through videoconferences and email. The *All of Us* data interface hosted by NIH has required the use of R and/or Python and IC faculty members often sought assistance with correcting errors in code, data wrangling and management, selecting appropriate statistical methods, and interpreting and visualizing data.

Another type of concern raised by participants involved the nature of the *All of Us* Researcher Workbench as a cloud computing platform. Many Academy participants, including faculty members, had little to no prior exposure to statistical computing using data stored in the cloud. Some members sought assistance with optimal data storage configuration, understanding costs associated with cloud computing, cloud connectivity issues, accessing relevant data to address research questions, and transferring R and/or Python coding knowledge to the Jupyter Notebooks cloud platform employed by the *All of Us* Research Program.

We selected our team of Academy coaches not only because of their technical abilities but also because of their willingness to engage researchers respectfully in a way that protected the researchers’ dignity and self-image. IC participants shared positive feedback in response. For example, 1 participant noted, “not only do we have professional development opportunities, but access to experts and coaches who assist us…the added value of being part of the Researcher Academy is really to provide support for our mentees.”

### Network support: quarterly newsletter

Our efforts to connect Academy participants and potential collaborators extended beyond webinars and events. We developed a quarterly digital newsletter called *Connections* featuring profiles of Academy-affiliated researchers, career guidance, and news about opportunities and events. Our opt-in subscriber list quickly grew to over 460 contacts, and our average open rate (approximately 53% through 2023) exceeded email marketing group Constant Contact’s “Research Services” industry average by more than 10 percent (see constantcontact.com). The newsletter’s focus on peer support and encouraging career skills may have contributed to robust content engagement.

### Network support: Faculty Scholar convenings

Throughout the first year, we hosted online convenings to offer dedicated space for sharing resources and opportunities specifically among faculty. Our Faculty Scholars Program offers a peer faculty network within and across IC groups. In future years, Senior Faculty Scholars who previously have participated will serve as experienced mentors to new Faculty Scholars.

## Course feedback and participant observations

During the first year of courses (September 2022 through June 2023), we solicited feedback from participants and assessed responses (*n* = 75 evaluations from 197 course registrations). Table 1 outlines responses from Year 1 course evaluations. Several themes emerged. First, tuition-free, online courses can provide researchers at institutions such as HBCUs useful technical knowledge. Virtually all participants (91%, or 68/75) completing an evaluation agreed that they learned a lot from the *All of Us* Researcher Academy course in which they participated. We also did not find evidence of discouragement or restrictive social norms at HBCUs regarding professional training. All participants taking a fall 2022 course on using R who completed an evaluation, for example, agreed that most people whose opinions they value would approve of their using the *All of Us* Researcher Workbench. Live interaction played an important role in the Academy experience, even in the case of an asynchronous course as the instructors offered live office hour sessions. Several participants noted appreciation for personal (online) interaction not only with course instructors and researchers at the ICs but also with Academy coaches to assist with analysis questions.

Qualitative comments offered by participants also suggested challenges facing researchers, however, including difficulties with scheduling. One participant noted, for example, “It is so hard to juggle time for the courses amidst all of our regular responsibilities.” Researchers at many HBCUs balance heavy teaching loads during each academic semester along with mentoring students and conducting research. Finding additional time for training and professional development can be daunting.

## Return of value for communities

The *All of Us* Researcher Academy has served both people not working at academic institutions and various communities by encouraging research which otherwise may not have occurred as well as by offering supports and avenues for professional development to a range of people. With *All of Us* Researcher Academy support, for example, faculty and student participants have investigated topics including disparities in healthcare utilization such as differences in postpartum depression treatment utilization as a function of racial and ethnic identity.^10^ Such research highlights important opportunities to encourage equity in treatment distribution. Elements of the *All of Us* Researcher Academy such as the inclusion of researchers affiliated with community colleges and the internship program introduce professional pathways into biomedical science and public health careers for students from a variety of socioeconomic backgrounds.

## Lessons learned for contexts beyond the *All of Us* Research Program

Certain resource conditions and considerations seem likely to facilitate efforts to build learning networks like the *All of Us* Researcher Academy. Organizers should identify an initial core group of relevant researchers based at potential partner institutions by investigating departmental directories and research proceedings before formally launching a network-building initiative, for example. Planning for continued engagement with network participants over time by establishing forums for connection and on-going coaching opportunities—rather than solely investing in a 1-time meeting—likely also is important for network success. Moreover, because the process of research and publication of that research typically is iterative and complex, organizers should set expectations for slow but meaningful growth in research productivity over time rather than anticipating publication outcomes overnight.

## Conclusions and future opportunities

By embracing a support model comprising explicit efforts to connect researchers at different institutions as well as opportunities for research guidance and feedback, the *All of Us* Researcher Academy has supported researchers’ journeys using the *All of Us* data set. However, the academy approach has required noteworthy costs, including the need to work within the schedules and constraints of participants and staffing to answer questions and address analysis concerns.

The *All of Us* Researcher Academy has offered relevant coursework, coaching, and connections to researchers at HBCUs by identifying peer-led model research and building forums in which researchers could build skills necessary to do similar work. Our approach could be extended to other types of institutions with a track record of supporting underrepresented students and researchers. Designing and implementing future learning networks, however, requires initial efforts to recruit a critical mass of researchers and dedicated staff time for nurturing and troubleshooting. Cross-institutional networks also require cross-institutional funding and organizational efforts, as well as time to establish and maintain trusted relationships that acknowledge historical inequity without stigmatizing those who have faced such inequity.

## Data Availability

Evaluation data reported in this article are available from the authors upon request.
